# Combination of Proteasome and Histone Deacetylase Inhibitors Overcomes the Impact of Gain-of-Function p53 Mutations

**DOI:** 10.1155/2018/3810108

**Published:** 2018-12-17

**Authors:** Xiangbing Meng, Shujie Yang, Yujun Li, Yiyang Li, Eric J. Devor, Jianling Bi, Xinhao Wang, Shaikamjad Umesalma, Dawn E. Quelle, William H. Thiel, Kristina W. Thiel, Kimberly K. Leslie

**Affiliations:** ^1^Department of Obstetrics and Gynecology, University of Iowa, Iowa City, Iowa 52242, USA; ^2^Holden Comprehensive Cancer Center, University of Iowa, Iowa City, Iowa 52242, USA; ^3^Department of Gynecology, The First Hospital of Jilin University, Changchun City, Jilin Province 130021, China; ^4^Department of Pharmacology, University of Iowa, Iowa City, Iowa 52242, USA; ^5^Department of Internal Medicine, University of Iowa, Iowa City, Iowa 52242, USA

## Abstract

Mutations in the “guardian of the genome” *TP53* predominate in solid tumors. In addition to loss of tumor suppressor activity, a specific subset of missense mutations confers additional oncogenic properties. These “gain-of-function” (GOF) mutations portend poor prognosis across cancer types regardless of treatment. Our objective in this study was to identify novel therapeutic opportunities to overcome the deleterious effects of GOF *TP53* mutants. Using gynecologic cancer cell lines with known *TP53* mutational status, we established that treatment with a proteasome inhibitor induced cell death in cells with two recurrent GOF *TP53* mutations (R175H and R248Q), and addition of a histone deacetylase inhibitor (HDACi) enhanced this effect. By contrast, p53-null cancer cells were relatively resistant to the combination. Proteasome inhibition promoted apoptosis of cells with *TP53* GOF mutations, potentially through induction of the unfolded protein response. In line with the reported hyperstabilization of GOF p53 protein, cells treated with HDACi exhibited reduced levels of p53 protein. Together, these data form the basis for future clinical studies examining therapeutic efficacy in a preselected patient population with GOF *TP53* mutations.

## 1. Introduction

The Cancer Genome Atlas (TCGA) project has substantiated the long-held notion that the “guardian of the genome” *TP53* is the most mutated gene in tumors [1]. Certain tumor types have an exceptionally high preponderance of mutations in *TP53*: for example, mutations in *TP53* occur in 96% of all serous ovarian tumors [2], and nearly all serous and ~25% of high-grade endometrioid endometrial cancers have mutations in *TP53* [3]. The prevalence of *TP53* mutations is also particularly high in head and neck cancer and breast cancer [1, 4].

While it is appreciated that *TP53* mutations occur in a substantial number of tumors, it is critically important to note that varying types of p53 mutant proteins exist, with different implications for chemosensitivity. Some mutations are relatively inconsequential from the perspective of p53 function, and proteins of this type retain wild-type activity. Other mutations are loss of function (LOF) or p53-null in which single amino acid changes completely inactivate or destabilize the protein. Finally, an interesting category is the gain-of-function (GOF) or “oncogenic” *TP53* mutations that convert p53 from a tumor suppressor to an oncogene. The majority of LOF and GOF *TP53* mutations result in loss of DNA binding to canonical p53 targets. However, GOF mutants also have new protein: protein interactions and/or transcriptional targets that confer an additional “oncogenic” functions [5–8]. To date, eight missense mutations in human *TP53* have been established as GOF mutations and result in the following amino acid changes: P151S, Y163C, R175H, L194R, Y220C, R248Q, R248W, R273C, R273H, R273L, and R282W.

Substantial clinical and preclinical data from a wide range of cancers demonstrate that GOF *TP53* mutations predict for poor response to treatment. In a recently published work, we evaluated the relationship of the eight GOF *TP53* mutations with progression-free survival (PFS), risk of recurrence, and response to standard platinum and taxane chemotherapy in serous ovarian cancer [9]. We found that 21.2% of serous ovarian cancer patients in TCGA cohort have a GOF *TP53* mutation, whereas 18.9% have LOF mutations [9]. Ovarian cancer patients with GOF *TP53* mutations have worse clinical outcomes compared to patients with unclassified *TP53* mutations (i.e., variants of unknown significance), including a shorter PFS and a 60% greater risk of recurrence [9]. These findings have important potential implications for all cancers characterized by mutations in *TP53.*

Analysis of *TP53* mutational status is now included in many next-generation sequencing tests. An obvious question, therefore, is how to convert these deadly oncogenic mutations into actionable mutations. Herein, we identify the combination of a proteasome inhibitor with an epigenetic modulator (histone deacetylase inhibitor (HDACi)) as a potent therapeutic strategy to overcome the deleterious effects of *TP53* GOF mutations. These preclinical data serve as the proof of concept for future trials evaluating specific combinatorial therapies in patients whose tumors contain *TP53* GOF mutations.

## 2. Materials and Methods

### 2.1. Reagents

All antibodies were purchased from Cell Signaling. Bortezomib, LBH589 (panobinostat), and MLN2238 (ixazomib) were purchased from Selleck Chemicals and suspended in DMSO.

### 2.2. Cell Lines and Culture Conditions

All cell lines used in this study were purchased from ATCC, except for Hec50 endometrial cancer cells that were kindly provided by Dr. Erlio Gurpide (New York University) as previously described [10]. Hec50 cells expressing R175H *TP53* GOF have been previously described [10]. All cell lines have been authenticated using STR analysis by biosynthesis.

### 2.3. Cell Viability Assays

Beginning 24 h after plating equal numbers of cells, cells were treated for 72 h followed by assessment of cell viability using the WST-1 assay per manufacturer's instructions (Clontech). Data were quantitated relative to values obtained for control (untreated) cells, which were set at 100% viability.

### 2.4. Western Blot Analysis

As previously described [10], cells were plated in 100 mm dishes and were allowed to grow for 24 h prior to treatment. After treatment, cells were harvested, lysed with extraction buffer (1% Triton X-100, 10 mM Tris-HCl pH 7.4, 5 mM EDTA, 50 mM NaCl, 50 mM NaF, 20 *μ*g/ml aprotinin, 1 mM PMSF, and 2 mM Na_3_VO_4_), and subjected to three freeze/thaw cycles. Equal amounts of protein (determined by the method of Bradford, BioRad) were subjected to SDS-PAGE followed by transfer to nitrocellulose membranes (BioScience). Membranes were probed with primary antibodies against cleaved caspase 3, Bip, *α*-tubulin, p53, p21, or *β*-actin followed by incubation with corresponding horseradish peroxidase-conjugated secondary antibody. The signal was visualized by chemiluminescence using ECL western blotting detection reagents (Pierce).

### 2.5. Statistical Analysis

All data were expressed as the mean ± SD. All statistical comparisons were performed using GraphPad Prism software. A *P* value < 0.05 was considered statistically significant.

## 3. Results

### 3.1. Sensitivity of Cancer Cells with Known p53 Status to Proteasome Inhibitors

**Figure 1:**
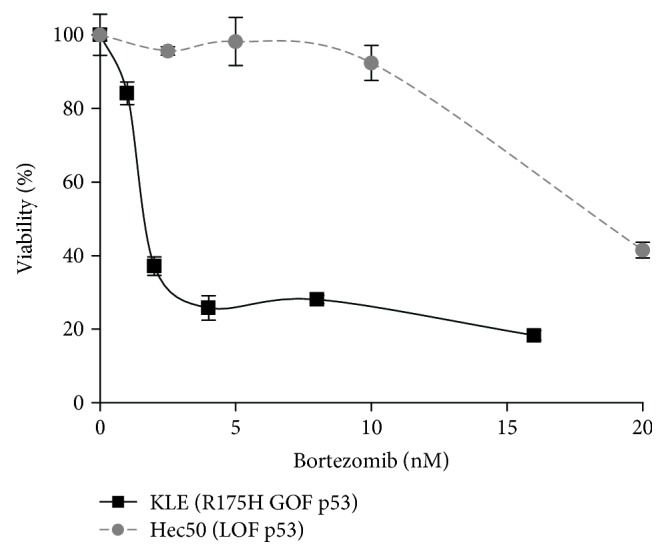
Proteasome inhibitor bortezomib induces massive cell killing in endometrial cancer cells with *TP53* GOF mutation R175H (KLE cells) but not LOF mutation (Hec50 cells). All experiments were performed three times. IC50: KLE cells, 2.1 ± 0.3 nM; Hec50 cells, 19.4 ± 1.0 nM; ^∗^*P* < 0.05 by Student's *t*-test.

We first examined the sensitivity of two well-characterized endometrial cancer cell lines with known p53 mutational status to the proteasome inhibitor, bortezomib (Velcade®). We made the unexpected discovery that KLE cells with the R175H GOF mutation were highly sensitive to the proteasome inhibitor bortezomib, whereas Hec50 cells with LOF p53 mutation were relatively resistant to bortezomib (Figure 1).

### 3.2. Addition of HDACi Enhances Sensitivity to Proteasome Inhibitor Treatment in Cells with Endogenous *TP53* GOF Mutations

**Figure 2:**
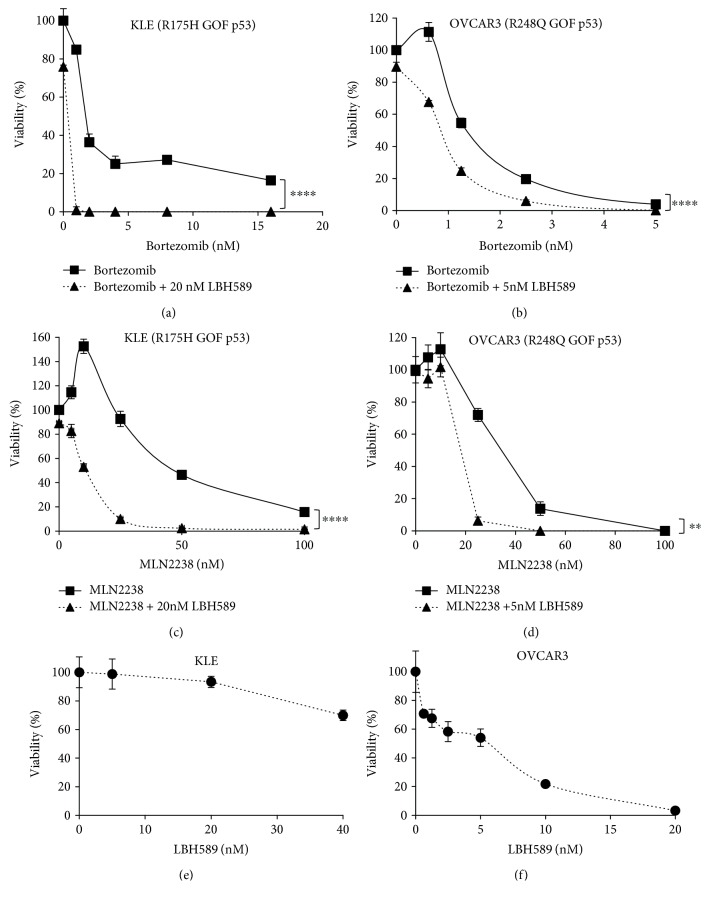
Gynecologic cancer cells with GOF *TP53* are highly sensitive to proteasome inhibitors alone or in combination with LBH589/panobinostat. Sensitivity (as measured by percent viability relative to untreated control) to bortezomib (a, b) or MLN2238/ixazomib (c, d) alone or in combination with LBH589/panobinostat was examined in KLE endometrial cancer cells with R175H GOF mutant (a, c) and OVCAR3 ovarian cancer cells with R248Q GOF mutant (b, d). The concentration of LBH589/panobinostat used in (a–d) was based on sensitivity to LBH589/panobinostat alone in KLE (e) and OVCAR3 (f) cells. All experiments were performed three times. ^∗∗^*P* < 0.01; ^∗∗∗∗^*P* < 0.0001 by two-way ANOVA with Sidak's multiple comparison test.

Next, we examined the impact of the addition of a histone deacetylase inhibitor. The combination of bortezomib with the HDACi LBH589 (panobinostat) further increased cell killing in KLE cells (R175H GOF) as compared to bortezomib alone (Figure 2(a)). Studies were also performed in the OVCAR3 ovarian cancer cell line that contains a different GOF *TP53* mutation, R248Q. Consistent with our findings in endometrial cancer cells, OVCAR3 cells were highly sensitive to bortezomib alone or in combination with HDACi (Figure 2(b)). The specific dose of LBH589/panobinostat was determined by assessing the sensitivity of each cell line to treatment with LBH589/panobinostat alone (Figures 2(e) and 2(f)). Since OVCAR3 cells contain a different *TP53* GOF mutation than KLE cells, these data suggest that the sensitivity to proteasome inhibition is not restricted to the R175H mutation.

Ixazomib (MLN2238) is a next-generation proteasome inhibitor that has replaced bortezomib in the clinic for multiple myeloma due to its improved activity and other characteristics, such as oral bioavailability [11, 12]. Therefore, we repeated the above experiments using ixazomib, either alone or in combination with the HDACi LBH589/panobinostat in KLE and OVCAR3 cells that express different *TP53* GOF mutations. Similar to the bortezomib studies, both KLE and OVCAR3 cells responded well to MLN2238/ixazomib (Figures 2(c) and 2(d)). Moreover, MLN2238/ixazomib was highly synergistic with the HDACi.

### 3.3. Exogenous Expression of GOF *TP53* in p53-Null Cells Sensitizes Cells to Proteasome Inhibitor + HDACi Therapy

**Figure 3:**
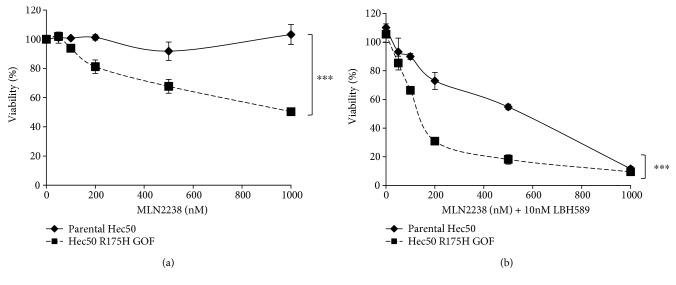
Sensitivity to MLN2283/ixazomib and LBH589/panobinostat combination treatment is dependent on the expression of GOF *TP53*. Sensitivity to MLN2238/ixazomib alone (a) or in combination with LBH589/panobinostat (b) was examined in parental Hec50 cells or Hec50 cells expressing the R175H GOF mutant. ^∗∗∗^*P* < 0.001 by two-way ANOVA with Sidak's multiple comparison test.

To further address the specific role of *TP53* GOF mutations in response to proteasome inhibitor + HDACi treatment, we introduced the p53 GOF mutant, R175H, in p53-null cells by exogenous expression [10]. As compared to parental cells, expression of p53R175H partially restored sensitivity to MLN2238/ixazomib (Figure 3(a)), and the addition of the HDACi LBH589 to the proteasome inhibitor backbone treatment substantially increased cell death.

**Figure 4:**
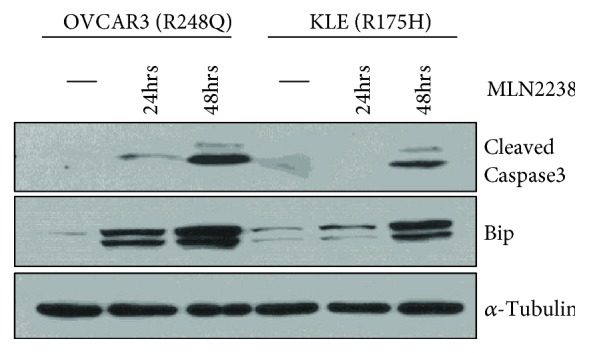
Treatment with MLN2238/ixazomib promotes apoptosis, potentially through the UPR pathway. Cells were treated for 0, 24, or 48 h with MLN2238/ixazomib and cell lysates analyzed by western blotting with the indicated antibodies (*α*-tubulin served as a loading control).

An established mechanism of action of proteasome inhibitors is the induction of cell death via apoptosis [13]. In both KLE and OVCAR3 cells, treatment with MLN2238/ixazomib promoted cleavage of caspase 3, a marker for apoptosis (Figure 4). Others have shown that proteasome inhibitors induce apoptosis by activating the unfolded protein response (UPR) pathway, a homeostatic mechanism that is normally triggered by accumulation of misfolded proteins in the endoplasmic reticulum [13]. A hallmark of the UPR pathway is increased expression of Bip/GRP78, a chaperone protein that induces proper folding of misfolded proteins such as GOF p53. Immunoblotting revealed that treatment with MLN2238/ixazomib increased the expression of Bip (Figure 4).

**Figure 5:**
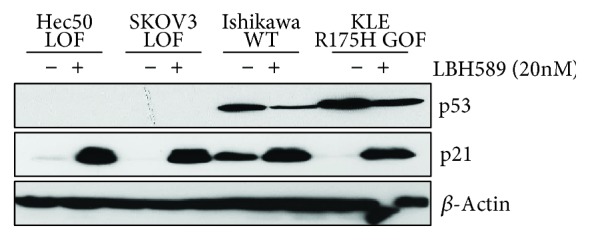
Treatment with LBH589/panobinostat reduces p53 protein levels. Cells lacking p53 (LOF) or expressing the indicated forms of p53 (WT or R175H GOF mutant) were treated with 20 nM panobinostat and levels of the indicated proteins measured by western blotting. p21 served as a positive control for HDACi activity. *β*-Actin served as a loading control.

Mutant p53 has also been shown to interact with histone deacetylases (e.g., HDAC2/6), which contributes to its stabilization and aberrant functions [14, 15]. Published evidence suggests that HDACi like LBH589/panobinostat may decrease the stability of mutant p53 [14, 15]. Consistent with these results, we found that treatment with LBH589/panobinostat caused a marked decrease in the total protein levels of p53 in KLE cells with the R175H GOF mutant (Figure 5). As a control for drug activity, we also examined p21 levels, which are known to be increased following treatment with HDACi regardless of p53 expression [16]. LBH589/panobinostat increased p21 in all cell lines examined (Figure 5).

## 4. Discussion

Despite clear data in multiple cancer types that *TP53* GOF mutations predict for poor outcomes, including resistance to therapy, to date no clinical trials have tested treatment strategies designed to specifically overcome the effects of *TP53* GOF mutations. In fact, *TP53* mutational status is widely ignored when making treatment decisions. Herein, we present a novel combinatorial strategy that effectively induces cell death specifically in cancer cells bearing GOF *TP53* mutations. Of note is that the combinatorial strategy of proteasome inhibitor plus HDACi was highly effective in cells with different recurrent *TP53* GOF mutations. We demonstrate this effect with two different proteasome inhibitors, bortezomib and ixazomib, indicating the potential generality of the approach. These data set the stage for future clinical studies in patients with GOF *TP53* mutations.

The cornerstone of personalized medicine is designing treatment strategies that overcome driver mutations in patients. However, mutations in *TP53* are not considered actionable in the traditional sense. One strategy to make *TP53* mutations druggable is based upon the principles of synthetic lethality, the term for a historical genetic observation that in the presence of certain single gene mutations, blocking or mutating a second gene leads to cell death, though neither mutation alone has a phenotype [17]. With respect to cancer therapy, synthetic lethality means capitalizing on the presence of a driver mutation to design novel treatments which block the compensatory survival pathways activated as a result of the mutation. To create therapeutic synthetic lethality, one must first know the driver mutation, understand the compensatory survival pathway that has been activated as a result of the mutation, and have an agent which can block this critical pathway. In a series of published studies, our group has established that treatment with a tyrosine kinase inhibitor (e.g., gefitinib, nintedanib, and cediranib) sensitizes p53-null cancer cells to paclitaxel-containing chemotherapy [10, 18, 19]. The mechanism is through abrogation of the G2/M cell cycle checkpoint. Enforcing the G2/M cell cycle checkpoint allows tumor cells to repair damaged DNA before entering mitosis, leading to chemoresistance [20–26]. Wild-type p53 normally maintains both the G1/S and G2/M checkpoints. However, emerging data suggest that p38MAPK can also maintain the G2/M checkpoint [27–29]. In cells with LOF p53, p38 is activated as an alternative means to maintain the G2/M checkpoint [25]. Therefore, treatment with an upstream agent that blocks p38 activation (e.g., tyrosine kinase inhibitors) sensitizes p53-null cells to paclitaxel, resulting in accumulation of cells in mitosis and massive cell death via mitotic catastrophe [10, 18].

Unfortunately, this same combinatorial strategy is not effective in cells with GOF p53. Specifically, our published data from cell models with endogenous and exogenous expression of GOF p53 mutants demonstrate that, in contrast to LOF p53, GOF forms of p53 constitutively enhance the G2/M checkpoint and are resistant to paclitaxel + tyrosine kinase inhibitors [10, 18]. Others have reported that p53 GOF mutants R175H, R273H, and R280K aberrantly induce p38 activation via transcriptional activation of MKK3 (an upstream kinase of p38), thereby maintaining the G2/M checkpoint [30]. Other established cancer therapeutics, such as temozolomide and tamoxifen, likewise are ineffective against tumor cells expressing *TP53* GOF mutants due to specific effects of mutant p53 on O6-methylguanine DNA-methyltransferase (MGMT) and estrogen receptor expression, respectively [31]. Therefore, alternative strategies are necessary to overcome the effects of GOF p53. One approach is to use small molecule drugs to restore the wild-type p53 conformation and thereby restore normal p53 anticancer function [31]. Our approach instead takes advantage of the unique properties of GOF p53 mutants, namely, aberrant folding and increased stability.

Here, we discovered that cells with GOF but not LOF *TP53* mutations are hypersensitive to proteasome inhibition, and addition of an HDACi (here, panobinostat) further enhanced cell killing. Both histone deacetylase inhibitors (vorinostat, panobinostat) and proteasome inhibitors (bortezomib, ixazomib) have been extensively studied in preclinical and clinical models of multiple cancer types [32]. Herein, we extend these prior findings to our cell models of ovarian and endometrial cancer, diseases for which new therapies are urgently needed.

Studies in multiple myeloma have provided significant mechanistic insight into why proteasome inhibitors are highly toxic to the cancer cells. For example, proteasome inhibition has been shown to promote apoptosis via terminal UPR [13]. Consistent with these data, we found that ixazomib treatment induced cleavage of caspase 3, a canonical marker of apoptosis, as well as expression of Bip/GPR78, a marker for ER stress. Since p53 GOF mutant protein is a misfolded protein, proteasome inhibition may induce cell death through excessive accumulation of misfolded proteins. Several studies have reported hyperstabilization of GOF p53 protein in cancer [33], which has been postulated to occur through more than one mechanism [14, 15, 34]. First, p53 GOF proteins are unable to bind the E3 ligase Mdm2, which negates the typical pathway of p53 ubiquitination and degradation via the proteasomal pathway [34]. Instead, p53 GOF protein is thought to be degraded by the lysosome in a process termed “chaperone-mediated autophagy” (CMA) [35]. Intriguingly, inhibition of the proteasome results in a compensatory induction of the activity of the CMA pathway [35]. Second, mutant p53 can be stabilized through interactions with heat shock proteins and histone deacetylases, and published evidence suggests that HDACi may decrease the stability of mutant p53 by disrupting its association with heat shock proteins [14, 15]. Therefore, one possibility is that HDACi potentiates the effects of the proteasome inhibitor by removing components of the chaperone complex, improving uptake in the lysosome, and leading to CMA-mediated p53 GOF degradation. Supporting this notion, we observed decreased total p53 protein levels upon treatment with panobinostat. In addition, histone deacetylase inhibitors have been shown to induce cell cycle arrest at the G1/S transition via upregulation of p21 [16], which we also demonstrate in cells with both wild-type (Ishikawa cells) and p53 GOF mutants (KLE, R175H p53). Whereas early studies with vorinostat suggested that G1/S cell cycle arrest is accomplished through upregulation of p53 [36], others have established that HDACi treatment destabilizes mutant p53, resulting in a marked decreased in p53 levels [14]. Our data are in line with the latter findings whereby treatment with LBH589/panobinostat resulted in a 50% or greater decrease in both GOF p53 (KLE cells) and wild-type p53 (Ishikawa cells).

Multiple myeloma is typified by accumulation of high levels of immunoglobulin, and thus the cells are extremely dependent upon proteasomal pathways for survival [37]. We speculate that GOF p53 mutants create a similar scenario that also necessitates a functional proteasome to maintain cell survival. Indeed, it has been suggested in the literature that excessive accumulation of mutant p53 may be more sensitive to proteasome and/or histone deacetylase inhibition [38]. Inhibiting the proteasome pathway would create a reliance on the lysosomal pathway for degrading the mutant p53, which is recognized as a misfolded protein. In line with this concept, we observed increased expression of Bip, a marker of the misfolded protein response pathway.

It is possible that distinct GOF mutations may differentially affect sensitivity to the single or combinatorial treatment regimen presented in this manuscript. In contrast to the findings presented herein, other studies have provided evidence that bortezomib sensitivity is dependent on wild-type p53 expression, whereby apoptosis is induced through p53-mediated downregulation of the prosurvival factor survivin [39, 40]. Cells that express a mutant p53 or p53-null cells were found to be resistant to bortezomib-induced apoptosis through sustained expression of survivin [40]. However, the specific GOF p53 mutants included in a previous study were R280K (MDA-MD-231 breast cancer cells) and E285K (RPMI-8226 multiple myeloma cells). While our data show similar results using cells expressing the R175H (Hec50 endometrial cancer cells) and R248Q (OVCAR3 ovarian cancer cells) mutants, a comprehensive analysis of cells expressing other recurrent p53 GOF mutants is warranted.

## 5. Conclusions

In summary, we present a novel therapeutic strategy for tumors with GOF *TP53* mutations using drugs that are already being advanced in clinical trials. These data suggest that p53 mutational status can be used as the foundation for defining personalized treatments.

## Data Availability

The data used to support the findings of this study are included within the article.
